# Circulating levels of leptin, adiposity and breast cancer risk

**DOI:** 10.1038/sj.bjc.6604913

**Published:** 2009-02-17

**Authors:** M-H Wu, Y-C Chou, W-Y Chou, G-C Hsu, C-H Chu, C-P Yu, J-C Yu, C-A Sun

**Affiliations:** 1 Graduate Institute of Life Sciences, National Defense Medical Center, Taipei, Taiwan, ROC; 2 School of Public Health, National Defense Medical Center, Taipei, Taiwan, ROC; 3 Department of Public Health, College of Medicine, Fu-Jen Catholic University, Taipei, Taiwan, ROC; 4 Department of Radiology, Tri-Service General Hospital, Taipei, Taiwan, ROC; 5 Headquarter, Tri-Service General Hospital, Taipei, Taiwan, ROC; 6 Department of Surgery, Tri-Service General Hospital, Taipei, Taiwan, ROC; 7 Department of Pathology, Tri-Service General Hospital, Taipei, Taiwan, ROC

**Keywords:** leptin, adiposity, breast cancer

## Abstract

The present case–control study was to investigate the relationships of plasma leptin level and anthropometric measures of adiposity with the risk of breast cancer. Questionnaire information, anthropometric measures and blood samples were taken before treatment from 297 incident cases with breast cancer and 593 controls admitted for health examination at the Tri-Service General Hospital, Taipei, between 2004 and 2006. Plasma levels of leptin were measured by RIA. Logistic regression analysis was used to estimate odds ratios (ORs) and 95% confidence intervals (CIs) for assessing the associations. Overall, higher leptin concentrations were significantly associated with an increased risk of breast cancer (OR (95% CI) for top *vs* bottom tertile of leptin was 1.63 (1.07–2.49), *P*_trend_=0.009). Waist circumference was a significant anthropometric factor for breast cancer in both pre- and postmenopausal women. Furthermore, the associations of leptin with breast cancer risk remained after adjustment for obesity indices. These results suggest that leptin may have an independent role in breast tumorigenesis. Regardless of the impact of circulating leptin, more research is needed to elucidate molecular mechanisms and local leptin levels that are critical for the development of breast cancers.

Epidemiologic evidence suggested that obesity is associated with an increased risk of breast cancer in women, primarily in postmenopausal population (Calle and Thun, 2004). However, the molecular mechanisms underlying the obesity–breast cancer link are not fully clear. One important factor contributing to obesity–breast carcinogenesis might be an excess exposure of mammary epithelium to various bioactive substances produced by the adipose tissue (adipokines). Indeed, adipose tissue is a source of oestrogens, insulin and insulin-like growth factors, all of which are believed to be involved in mammary tumorigenesis (Schäffler *et al*, 2007). The most prominent adipokine is leptin, which is best known as a regulator of food intake and energy balance in the hypothalamus (Auwerx and Staels, 1998). Interestingly, leptin is also implicated in the regulation of reproductive hormones and function (Zhang *et al*, 2005). Notably, leptin consistently stimulates the proliferation of benign and malignant epithelial breast cells *in vitro* as measured by DNA synthesis and upregulation of downstream regulators of cellular proliferation (Dieudonne *et al*, 2002; Hu *et al*, 2002). Thus, the intriguing possibility exists that leptin could be directly related to breast carcinogenesis by underlying the effects of obesity on cancer development. We have undertaken a case–control study to test this hypothesis.

## Materials and methods

### Case and control selection

This case–control study was conducted at the Tri-Service General Hospital, Taipei, Taiwan, from January 2004 to November 2006. To account for the notions that type 2 diabetes and metabolic syndrome (MS) have been associated with increased serum leptin levels and elevated risk of breast cancer (Vona-Davis *et al*, 2007), individuals with a history of type 2 diabetes and/or MS were excluded from this study. On the basis of the hospital chart number, the cases involved 297 women consecutively selected from subjects with a first confirmed histopathologic diagnosis of breast carcinoma in the age range of 24–72 years. The histopathological profile included 216 cases of invasive ductal carcinoma, 31 cases of mucinous carcinoma or invasive lobular carcinoma and 50 cases of carcinoma *in situ*. There were 172 premenopausal and 125 postmenopausal cases with breast cancer. Control subjects comprising individuals without a history of cancer were simultaneously recruited from the health examination clinics of the same hospital during the same study period. Two control subjects were matched to each case by date of enrolment (±3 months) and duration of fasting (±4 h). One control subject with inadequate specimen was excluded, resulting in 297 cases and 593 controls included in this study.

## Collection of questionnaire data and blood specimens

Once case patients and control subjects agree to participate, written informed consent was obtained from all the subjects. The research protocol was approved by the Institutional Review Board at the Tri-Service General Hospital, Taipei. All participants underwent personal interview administered by well-trained interviewers in conformance with institutional guidelines for studies including human subjects. Data were collected on sociodemographic characteristics, menstrual and reproductive history, menopausal status, lifestyle behaviours and medical history as well as family history of breast and other cancers. More specifically, in this study, menopausal status was defined as last menstruation after 1 year free of menstrual cycle, and no attempt was made to distinguish between women with artificial and those with natural menopause. Immediately after the interview, a 10-ml blood sample was drawn into coded EDTA-treated tubes and centrifuged at 1467 *g* for 10 min at room temperature within 10 h of collection. Plasma, buffy coat and red blood cells were separated and stored at −70°C until subsequent analysis. In this study, efforts had been made to obtain questionnaire data and biospecimens before cases' acceptance with surgery and/or receiving adjuvant therapy, any influence of treatment protocol being unlikely.

### Anthropometric measurements and laboratory analysis of plasma leptin levels

Participants reported information regarding height and weight. Body mass index (BMI), as an indicator of generalised obesity, was calculated as weight in kilograms divided by the square of height in metres. In addition, the measurements of waist and hip circumferences were performed by trained clinical staff using standardised techniques. Waist girth was determined with a measuring tape placed horizontally around the midpoint between the iliac crest and lower margin of the ribs. Hip girth was the maximum circumference around the buttocks posteriorly and the symphysis pubis anteriorly. As a result, waist circumference (WC) and waist-to-hip ratio (WHR) represent a measure of central adiposity. Plasma leptin concentrations were measured in a single run using a commercially available RIA kit (Linco Research Inc., Missouri, MO, USA) according to the manufacturer's instructions. The sensitivity of this assay is 0.5 ng ml^−1^; the intraassay coefficient of variation being 4.98% and the interassay coefficient of variation being 4.5%. Leptin is extremely stable and can be stored at −20°C for prolonged periods of time and even at 4°C or room temperature for several days without significant degradation (Ma *et al*, 1996). All matched case–control blood samples were handled identically and assayed in the same analytical run. The blood samples were labelled by number only and ordered randomly within each case–control pair. Accordingly, laboratory personnel were unaware of the case–control status.

### Statistical analysis

Differences between cases and controls in age at menarche, age at first full-term pregnancy (FFTP), age at menopause and parity numbers were tested using the Student's *t*-test. Spearman correlation coefficients were used to examine cross-sectional relationships between leptin and anthropometric measures of adiposity. In addition, conditional logistic regression, which preserves the matching of cases and controls, was used to estimate odds ratios (ORs) and 95% confidence intervals (CIs) for the associations between plasma leptin levels and anthropometric measures and breast cancer risk.

## Results

The baseline characteristics of cases and controls are summarised in Table 1 . The mean age (±s.d.) of the cases and controls was 49.7 (±8.7) and 48.7 (±8.5) years, respectively. There were no statistically significant differences between cases and controls in terms of age at menarche (13.6 *vs* 13.7 years), age at FFTP (26.7 *vs* 26.5 years), age at menopause (48.9 *vs* 48.5 years) and parity number (2.0 *vs* 2.0). By contrast, there were statistically significant differences between cases and controls for anthropometric measures of adiposity. The average BMI, WC and WHR were higher in breast cancer cases than in control subjects (23.4 *vs* 22.9 kg m^−2^, *P*=0.0648; 77.7 *vs* 75.1 cm, *P*<0.0001; and 0.81 *vs* 0.78, *P*<0.0001, respectively).

To assess the relationship of plasma leptin levels with anthropometric measures of adiposity, we performed a correlation analysis among control subjects. As expected, plasma leptin was significantly positively correlated with BMI (*r*=0.59, *P*<0.0001), WC (*r*=0.50, *P*<0.0001) and WHR (*r*=0.23, *P*<0.0001). The mean (±s.d.) levels of leptin in cases with invasive breast cancer, patients with carcinoma *in situ* and control subjects were 10.4(±7.0), 8.7(±5.3) and 8.4 (±5.3 ng ml^−1^), respectively. We further categorised plasma leptin either as high or as low levels (based on the 75 percentile in the control group) or into three groups (based on the tertile values in the control group) when appropriate for subsequent analyses. Table 2 presents the risk of breast cancer in relation to plasma leptin levels. Overall, higher leptin concentrations were statistically significantly associated with an increased risk of breast cancer. Compared with those in the lowest tertile, the ORs of breast cancer for women in the second and third tertiles were 0.93 (95% CI=0.63–1.38) and 1.63 (95% CI=1.07–2.49), respectively (*P*_trend_=0.0091). In subgroup analyses according to menopausal status, a similar pattern regarding breast cancer risk associated with higher levels of leptin was observed in postmenopausal women. Additional adjustment for BMI and WC slightly attenuated these associations. Restricting analysis to cases with invasive cancers and control subjects did not reveal any other remarkable discrepancies in results (data not shown). Concerning the roles of general and central obesity, we found that increasing BMI values were associated with reduced risk of breast cancer in premenopausal women after adjustment for circulating leptin levels (OR=0.22; 95% CI=0.10–0.48 for the highest *vs* the lowest tertile; *P*_trend_=0.001), whereas an increasing WC was associated with increased risk of breast cancer in both pre- and postmenopausal women when an adjustment for leptin concentrations was made (ORs (95% CI) for the highest *vs* the lowest tertile in pre- and postmenopausal women were 3.54 (1.55–8.10) and 2.85 (1.19–6.84), respectively). Accordingly, WC was a significant anthropometric factor for breast cancer. Thus, we further evaluate the possible joint effect of WC and leptin on breast cancer risk for the whole group and separately for pre- and postmenopausal women (Table 3 ). In general, the highest risk was observed for those who had higher WC measures and elevated circulating leptin levels. When compared with risks for women with WC measures and leptin concentrations less than the third tertiles, the ORs (95% CI) for those who had WC measures and plasma leptin levels in the highest tertiles for the whole group and separately for pre- and postmenopausal women were 2.51 (1.51–4.19), 1.99 (1.06–3.39) and 3.25 (1.53–6.91), respectively.

## Discussion

Leptin is a peptide hormone that is mainly synthesised and secreted by the adipose tissue (Considine *et al*, 1996; Auwerx and Staels, 1998). The plasma leptin concentrations increased in direct proportion to the adipose mass (Considine *et al*, 1996; Zhang *et al*, 2005). Although leptin has been viewed as the hormonal signal for the regulation of energy homoeostasis (Auwerx and Staels, 1998), several lines of evidence suggest that leptin plays a much broader physiological role (Rosenbaum and Leibel, 1999; Zhang *et al*, 2005). On a cellular level, leptin has been found to act as a metabolic regulator and motogenic and pro-angiogenic factors (Sierra-Honignann *et al*, 1998; Hardwick *et al*, 2001; Hu *et al*, 2002; Chio *et al*, 2004; Yin *et al*, 2004; Chen *et al*, 2006a). In addition, new evidence suggests that leptin could be involved in tumorigenesis, especially in the development of breast, colorectal and prostate cancers (Garofalo and Surmacz, 2006).

In this study, we found a significant increase in the risk of breast cancer associated with a high plasma level of leptin. Intriguingly, women in the intermediate tertile of leptin had a nonsignificantly reduced risk for breast cancer compared with those in the lowest tertile. The reasons for this observation are unclear and may reflect the play of chance. Although leptin has been shown to be correlated with breast cancer risk in this study and earlier reports (Tessitore *et al*, 2004; Han *et al*, 2005; Chen *et al*, 2006b; Hou *et al*, 2007; Liu *et al*, 2007), contradictory results were also documented by other investigators. Some reports suggested a negative correlation between leptin and breast cancer in the premenopausal but not postmenopausal group (Petridou *et al*, 2000), whereas several authors described that there was no significant association between circulating leptin and breast cancer risk (Mantzoros *et al*, 1999; Coskun *et al*, 2003; Sauter *et al*, 2004; Stattin *et al*, 2004; Woo *et al*, 2006). The inconsistent data obtained with circulating leptin and breast cancer risk could be, at least in part, explained by differences in sample preparation and measurement techniques as well as the lack of control for potential factors that influence leptin concentrations, such as food intake. Indeed, the hypothesis that leptin is mechanistically related to the development of breast cancer is supported by several breast cancer cell models showing that leptin induces proliferation, survival and anchorage-independent growth (Dieudonne *et al*, 2002; Hu *et al*, 2002; Yin *et al*, 2004). Furthermore, leptin has been found to modulate both oestrogen synthesis and oestrogen receptor-*α* activity. For instance, leptin can upregulate the aromatase gene expression and aromatase activity in MCF-7 cells, possibly leading to an increased oestrogen synthesis (Catalano *et al*, 2004). The relevance of leptin signalling in breast tumorigenesis is reinforced by the observation that both leptin and leptin receptor appear to be significantly overexpressed in breast cancer tissue relative to non-cancer epithelium (Ishikawa *et al*, 2004). Despite the large body of *in vitro* data suggesting the role of leptin in breast tumorigenesis, there was a large gap between the mean serum leptin concentration in experiments (above 100 ng ml^−1^) and in that of epidemiological studies (range: 8–41 ng ml^−1^). Furthermore, regardless of the impact of circulating leptin, recent studies suggest that carcinogenesis could be induced by an overabundance of locally produced leptin. The analysis of approximately 300 biopsies revealed that leptin is overexpressed in breast cancer, whereas it is absent or expressed at very low levels in normal epithelium or benign tumours (Ishikawa *et al*, 2004; Garofalo *et al*, 2006). Taken together, as leptin has been associated with cancer risk, but has not been shown to be oncogenic, further studies are needed to elucidate the levels of leptin that are needed in human breast tissue for the development of cancer.

Numerous studies have established that obesity is a risk factor for breast cancer development (Stoll, 1994; Calle and Thun, 2004). Specifically, the relationship between obesity assessed by BMI and breast cancer risk differs between pre- and postmenopausal women. Body mass index has a strong positive association with postmenopausal breast cancer and an inverse correlation with premenopausal cancer risk (van den Brandt *et al*, 2000). However, central obesity, mainly measured by the WC or WHR, can increase breast cancer risk in both premenopausal and postmenopausal populations (Calle and Thun, 2004). In this study, women with breast cancer appeared to have high BMI, WC and WHR than control subjects (Table 1). When stratification by menopausal state and adjustment for plasma leptin concentration was made, results suggest that general obesity assessed by BMI and central obesity measured by WC may both have effects on breast cancer risk. General obesity has an inverse effect in premenopausal women, whereas central obesity appears to be positively associated with increased risk of breast cancer in both pre- and postmenopausal women. It has been noted that Asian women have greater amounts of visceral fat – the adipose tissue component most strongly associated with insulin resistance and lower sex hormone-binding globulin – for a given waist measurement than Caucasian women (Lovejoy *et al*, 1996). As a result, central obesity may have an important role in breast tumorigenesis in Chinese women (Ng *et al*, 1997).

It is worth noting that the association between plasma leptin concentrations and breast cancer risk remained after adjustment for obesity indices in this study. In other words, the positive associations with leptin level were independent of measures of adiposity (Table 2), and obesity and leptin appear to have independent roles in breast carcinogenesis (Table 3), as our earlier observation on adiponectin–breast cancer association (Tian *et al*, 2007). These results seem to suggest a possibility that other growth hormones may contribute to obesity–cancer link and mechanisms other than energy balance may be involved.

The limitations of our study design warrant discussion. There was only one-time measurement of leptin concentration in this study. However, a single measurement of circulating leptin levels appears to reflect an individual's long-term levels of leptin quite well, because even in samples taken 4 years apart, intraclass correlation was high (*r*=0.74) (Chu *et al*, 2001). Meanwhile, any random error in the assays would tend to underestimate the true association. In addition, a recent study has shown that leptin was produced by breast cancer cells (Tessitore *et al*, 2000), and it is possible that levels of circulating leptin in subjects for whom samples were collected subsequent to breast cancer diagnosis and/or receiving adjuvant treatments may be influenced by the disease process and/or treatment protocol. Although we restricted our case–control comparisons to patients whose blood was drawn before surgery and/or receiving adjuvant therapy, results must be viewed cautiously. On the other hand, Chen *et al* (2006b) compared serum leptin levels in patients with breast cancer, before surgery and 1 month after surgery, and found no significant difference between the two groups. This observation suggested that breast cancer cells produce only small amount of the circulating levels of leptin and other body adipose tissues might be the major contributor. The current report has other limitations that the effects of premenopausal menstrual cycle and postmenopausal hormone replacement therapy (HRT) on leptin concentrations were not directly assessed. Circumstantial evidence suggests that ovarian hormones affect leptin production. Leptin is reported to fluctuate with the menstrual cycle (Shimizu *et al*, 1997), and premenopausal HRT may regulate leptin concentrations (Augoulea *et al*, 2005). However, our data indicated that there was no significant difference in the average duration between the date of last menstruation and the date of enrolment between premenopausal cases and controls (25.5 *vs* 26.5 days), and this average duration was not significantly associated with circulating leptin levels (the Spearman correlation coefficient in the control group was 0.0823; *P*=0.8361). In addition, our data showed that there was no significant difference in leptin concentrations between women who received postmenopausal HRT (51 cases and 145 controls) and those who were non-HRT users (74 cases and 114 controls) (8.78 *vs* 9.25 ng ml^−1^, *P*=0.810). Thus, the phase of menstrual cycle in premenopausal women and HRT in postmenopausal women may not affect the case–control comparisons for leptin concentrations in this study.

In conclusion, the converging lines of evidence from this study and earlier *in vitro* studies provide the background biological context for considering a role for leptin in the pathogenesis of breast cancer. Further investigation is warranted to confirm and expand on this empirical finding.

## Figures and Tables

**Table 1:** Characteristics of breast cancer cases and their matched controls

**Baseline characteristics**	**Cases (*n*=297)**	**Controls (*n*=593)**	***P*-value**
Age (year)	49.66±8.72	48.71±8.51	0.1196
Age at menarche (year)	13.64±1.50	13.67±1.53	0.7624
Age at FFTP (year)	26.72±4.14	26.46±4.05	0.4049
Age at menopause (year)	48.89±5.15	48.48±4.82	0.4466
Number of parity	2.01±1.15	2.02±1.08	0.9314
BMI (kg m^−2^)	23.36±4.00	22.88±2.93	0.0648
Waist circumference (cm)	77.67±9.37	75.13±7.56	<0.0001
WHR	0.81±0.06	0.78±0.05	<0.0001

BMI=body mass index; FFTP=first full-term pregnancy; WHR=waist-to-hip ratio.

**Table 2:** Breast cancer risk associated with plasma leptin concentrations

**Leptin level (ng ml^−1^)**	**No. of cases (%)**	**No. of controls (%)**	**Matched OR^a^ (95% CI)**	**Adjusted OR^b^ (95% CI)**
*All women*				
<5.06	68 (22.9)	147 (24.9)	1.00 (reference)	1.00 (reference)
5.06–10.90	124 (41.8)	299 (50.8)	0.93 (0.63–1.38)	0.87 (0.60–1.26)
>10.90	105 (35.3)	143 (24.3)	1.63 (1.07–2.49)	1.58 (1.02–2.43)
*P*_trend_			0.009	0.039
				
*Premenopausal*				
<4.82	37 (21.5)	83 (25.0)	1.00 (reference)	1.00 (reference)
4.82–10.60	83 (48.3)	168 (50.6)	1.11 (0.68–1.75)	0.99 (0.61–1.61)
>10.60	52 (30.2)	81 (24.4)	1.40 (0.83–2.38)	1.63 (0.91–2.89)
*P*_trend_			0.200	0.103
				
*Postmenopausal*				
<5.20	28 (22.4)	34 (24.9)	1.00 (reference)	1.00 (reference)
5.20–11.50	47 (37.6)	131 (50.9)	0.78 (0.44–1.37)	0.71 (0.40–1.26)
>11.50	50 (40.0)	62 (24.2)	1.69 (0.95–3.06)	1.35 (0.70–2.60)
*P*_trend_			0.050	0.324

CI=confidence interval; OR=odds ratio.

a Odds ratios were matched on the date of enrolment and fasting status.

b In addition to matched variables, odds ratios were also adjusted for body mass index and waist circumference.

**Table 3:** Joint effect of WC status and plasma leptin levels on breast cancer risk

**Group**	**WC^a^ (cm)**	**Leptin^a^ (ng ml^−1^)**	**No. of cases (%)**	**No. of controls (%)**	**OR^b^ (95% CI)**	***P*_trend_**
All women						
	⩽80	⩽10.9	153 (51.9)	375 (63.8)	1.00 (reference)	
	⩽80	>10.9	45 (15.2)	65 (11.0)	2.00 (1.27–3.17)	
	>80	⩽10.9	38 (12.9)	71 (12.1)	1.65 (0.99–2.76)	
	>80	>10.9	59 (20.0)	77 (13.1)	2.51 (1.51–4.19)	0.0006
Premenopausal						
	⩽80	⩽10.6	110 (64.0)	214 (64.5)	1.00 (reference)	
	⩽80	>10.6	25 (14.5)	35 (10.5)	1.90 (0.98–4.02)	
	>80	⩽10.6	10 (5.8)	37 (11.1)	1.29 (0.59–2.83)	
	>80	>10.6	27 (15.7)	46 (13.9)	1.99 (1.06–3.39)	0.0572
Postmenopausal						
	⩽80	⩽11.5	47 (38.2)	149 (58.0)	1.00 (reference)	
	⩽80	>11.5	15 (12.2)	30 (11.7)	1.64 (0.77–3.53)	
	>80	⩽11.5	27 (22.0)	46 (17.9)	1.85 (0.91–3.75)	
	>80	>11.5	34 (27.6)	32 (12.4)	3.25 (1.53–6.91)	0.0029

CI=confidence interval; OR=odds ratio; WC=waist circumference.

a WC status and plasma leptin were categorised by the third tertile value in the control group.

b In addition to matched variables, odds ratios were also adjusted for body mass index.

## References

[bib1] Augoulea A, Mastorakos G, Lambrinoudaki I, Christodoulakos G, George C (2005) Role of postmenopausal hormone replacement therapy on body fat gain and leptin levels. Gynecol Endocrinol 20: 227–235 16019366 10.1080/09513590400027372

[bib2] Auwerx J, Staels B (1998) Leptin. Lancet 351: 737–742 9504534 10.1016/S0140-6736(97)06348-4

[bib3] Calle EE, Thun MJ (2004) Obesity and cancer. Oncogene 23: 6365–6378 15322511 10.1038/sj.onc.1207751

[bib4] Catalano S, Mauro L, Marsico S, Giordano C, Rizza P, Rago V, Montanaro D, Maggiolini M, Panno ML, Ando S (2004) Leptin induces, via ERK1/ERK2 signal, functional activation of estrogen receptor *α* in MCF-7 cells. J Biol Chem 279: 19908–19915 14985328 10.1074/jbc.M313191200

[bib5] Chen C, Chang YC, Liu CL, Chang KJ, Guo IC (2006a) Leptin – induced growth of human ER-75-1 breast cancer cells is associated with up-regulation of cyclin D1 and c-Myc and down-regulation of tumor suppressor p53 and p21 WAF1/CIP1. Breast Cancer Res Treat 98: 121–132 16752079 10.1007/s10549-005-9139-y

[bib6] Chen DC, Chung YF, Yeh YT, Chaung HC, Kuo FC, Fu OY, Chen HY, Hou MF, Yuan SF (2006b) Serum adiponectin and leptin levels in Taiwanese breast cancer patients. Cancer Lett 237: 109–114 16019138 10.1016/j.canlet.2005.05.047

[bib7] Chio JH, Park SH, Leung PC, Chio KC (2004) Expression of leptin receptors and potential effects of leptin on the cell growth and activation of mitogen-activated protein kinases in ovarian cancer cells. J Clin Endocrinol Metab 90: 207–210 15522945 10.1210/jc.2004-0297

[bib8] Chu NF, Spiegelman D, Hotamisligil GS, Rifai N, Stampfer M, Rimm EB (2001) Plasma insulin, leptin, and soluble TNF receptors levels in relation to obesity-related atherogenic and thrombogenic cardiovascular disease risk factors among men. Atherosclerosis 157: 495–503 11472752 10.1016/s0021-9150(00)00755-3

[bib9] Considine RV, Sinha MK, Heiman ML, Kriauciunas A, Stephens TW, Nyce MR, Ohannesian JP, Marco CC, McKee LJ, Bauer TL, Caro JF (1996) Serum immunoreactive-leptin concentrations in normal-weight and obese humans. N Engl J Med 341: 913–915 10.1056/NEJM199602013340503 8532024

[bib10] Coskun U, Gunel N, Toruner FB, Sancak B, Onuk E, Bayram O (2003) Serum leptin, prolactin and vascular endothelial growth factor (VEGF) levels in patients with breast cancer. Neoplasma 50: 41–46 12687277

[bib11] Dieudonne MN, Machinal-Quelin F, Serazin-Leory V, Leneveu MC, Pecquery R, Giudicelli Y (2002) Leptin mediates a proliferative response in human MCF7 breast cancer cells. Biochem Biophys Res Commun 293: 622–628 12054648 10.1016/S0006-291X(02)00205-X

[bib12] Garofalo C, Koda M, Cascio S, Sulkowska M, Kanczuga-Koda L, Golaszewska J, Russo A, Sulkowski S, Surmacz E (2006) Increased expression of leptin and leptin receptors as a marker of breast cancer progression: possible role of obesity-related stimuli. Clin Cancer Res 12: 1447–1453 16533767 10.1158/1078-0432.CCR-05-1913

[bib13] Garofalo C, Surmacz E (2006) Leptin and cancer. J Cell Physiol 207: 12–22 16110483 10.1002/jcp.20472

[bib14] Han C, Zhang HT, Du L, Liu X, Jing J, Zhao X, Yang X, Tian B (2005) Serum levels of leptin, insulin, and lipids in relation to breast cancer in China. Endocrine 26: 19–24 15805581 10.1385/ENDO:26:1:019

[bib15] Hardwick JC, van den Brink GR, Offerhaus GJ, van Deventer SJ, Peppelenbosch MP (2001) Leptin is a growth factor for colonic epithelial cells. Gastroenterology 121: 79–90 11438496 10.1053/gast.2001.25490

[bib16] Hou WK, Xu YX, Yu T, Zhang L, Zhang WW, Fu CL, Sun Y, Wu Q, Chen L (2007) Adipocytokines and breast cancer risk. Chin Med J 120: 1592–1596 17908478

[bib17] Hu X, Juneza SC, Maihle NJ, Cleary MP (2002) Leptin—a growth factor in normal and malignant breast cells and for normal mammary gland development. J Natl Cancer Inst 94: 1704–1711 12441326 10.1093/jnci/94.22.1704

[bib18] Ishikawa M, Kitayama J, Nagawa H (2004) Enhanced expression of leptin and leptin receptor (OB-R) in human breast cancer. Clin Cancer Res 10: 4325–4331 15240518 10.1158/1078-0432.CCR-03-0749

[bib19] Liu CL, Chang YC, Cheng SP, Chern SR, Yang TL, Lee JJ, Guo IC, Chen CP (2007) The roles of serum leptin concentration and polymorphism in leptin receptor gene at codon 109 in breast cancer. Oncology 72: 75–81 18004080 10.1159/000111097

[bib20] Lovejoy JC, de la Bretonne JA, Klemperer M, Tulley R (1996) Abdominal fat distribution and metabolic risk factors: effect of race. Metabolism 45: 1119–1124 8781299 10.1016/s0026-0495(96)90011-6

[bib21] Ma Z, Gingerich RL, Santiago JV, Klein S, Smith CH, Landt M (1996) Radioimmunoassay of leptin in human plasma. Clin Chem 42: 942–946 8665687

[bib22] Mantzoros CS, Bolhke K, Moschos S, Cramer DW (1999) Leptin in relation to carcinoma *in situ* of the breast: a study of premenopausal cases and controls. Int J Cancer 380: 523–526 10.1002/(sici)1097-0215(19990209)80:4<523::aid-ijc7>3.0.co;2-c 9935151

[bib23] Ng EH, Gao F, Ji CY, Ho GH, Soo KC (1997) Risk factors for breast carcinoma in Singaporean Chinese women: the role of central obesity. Cancer 80: 725–731 9264356 10.1002/(sici)1097-0142(19970815)80:4<725::aid-cncr11>3.0.co;2-v

[bib25] Petridou E, Papadiamantis Y, Markopoulos C, Spanos E, Dessypris N, Trichopoulos D (2000) Leptin and insulin growth factor I in relation to breast cancer. Cancer Causes Control 11: 383–388 10877331 10.1023/a:1008903727238

[bib24] Rosenbaum M, Leibel RL (1999) The role of leptin in human physiology. N Engl J Med 341: 913–915 10486426 10.1056/NEJM199909163411211

[bib26] Sauter ER, Garofalo C, Hewett J, Hewett JE, Morelli C, Surmacz E (2004) leptin expression in breast nipple aspirate fluid (NAF) and serum is influenced by body mass index (BMI) but not by the presence of breast cancer. Horm Metab Res 36: 336–340 15156414 10.1055/s-2004-814490

[bib27] Schäffler A, Schölmerich J, Buechler C (2007) Mechanisms of disease: adipokines and breast cancer – endocrine and paracrine mechanisms that connect adiposity and breast cancer. Nat Clin Pract Endocrinol Metab 3: 345–354 17377617 10.1038/ncpendmet0456

[bib30] Shimizu H, Shimomura Y, Nakanishi Y, Futawari T, Ohtani K, Sato N, Mori M (1997) Estrogen increases *in vivo* leptin production in rats and human subjects. J Endocrinol 154: 285–292 9291839 10.1677/joe.0.1540285

[bib28] Sierra-Honignann M, Nath A, Murakami C, Garcia-Cardena G, Papapetropoulos A, Sessa W, Madge LA, Schechner JS, Schwabb MB, Polverini PJ, Flores-Riveros JR (1998) Biological action of leptin as an angiogenic factor. Science 281: 1683–1686 9733517 10.1126/science.281.5383.1683

[bib31] Stattin P, Soderberg S, Biessy C, Lenner P, Hallmans G, Kaaka R, Olsson T (2004) Plasma leptin and breast cancer risk: a prospective study in northern Sweden. Breast Cancer Res Treat 86: 191–196 15567935 10.1023/B:BREA.0000036782.11945.d7

[bib29] Stoll BA (1994) Breast cancer: the obesity connection. Br J Cancer 69: 799–801 8180007 10.1038/bjc.1994.157 PMC1968889

[bib32] Tessitore L, Vizio B, Jenkins O, De Stefano I, Ritossa C, Argiles JM, Benedetto C (2000) Leptin expression in colorectal and breast cancer patients. Int J Mol Med 5: 421–426 10719061 10.3892/ijmm.5.4.421

[bib33] Tessitore L, Vizio B, Pesola D, Cecchini F, Mussa A, Argiles JM, Benedetto C (2004) Adipocyte expression and circulating levels of leptin increase in both gynaecological and breast cancer patients. Int J Oncol 24: 1529–1535 15138597

[bib34] Tian YF, Chu CH, Wu MH, Chang CL, Yang T, Chou YC, Hsu GC, Yu CP, Yu JC, Sun CA (2007) Anthropometric measures, plasma adiponectin, and breast cancer risk. Endocr Relat Cancer 14: 669–677 17914097 10.1677/ERC-06-0089

[bib35] van den Brandt PA, Spiegelman D, Yaun SS, Adami HO, Beeson L, Folsom AR, Fraser G, Goldbohm RA, Graham S, Kushi L, Marshall JR, Miller AB, Rohan T, Smith-Warner SA, Speizer FE, Willett WC, Wolk A, Hunter DJ (2000) Pooled analysis of prospective cohort studies on height, weight, and breast cancer risk. Am J Epidemiol 152: 514–527 10997541 10.1093/aje/152.6.514

[bib36] Vona-Davis L, Howard-McNatt M, Rose DP (2007) Adiposity, type 2 diabetes and the metabolic syndrome in breast cancer. Obes Rev 8: 395–408 17716297 10.1111/j.1467-789X.2007.00396.x

[bib37] Woo HY, Park H, Ki CS, Park YL, Bae WG (2006) Relationships among serum leptin, leptin receptor gene polymorphisms, and breast cancer in Korea. Cancer Lett 237: 137–142 16011872 10.1016/j.canlet.2005.05.041

[bib38] Yin N, Wang D, Zhang H, Yi X, Sun X, Shi B, Wu H, Wu G, Wang X, Shang Y (2004) Molecular mechanisms involved in the growth stimulation of breast cancer cells by leptin. Cancer Res 64: 5870–5875 15313931 10.1158/0008-5472.CAN-04-0655

[bib39] Zhang F, Chen Y, Heiman M, Dimarchi R (2005) Leptin: structure, function and biology. Vitam Horm 71: 345–372 16112274 10.1016/S0083-6729(05)71012-8

